# Statins accelerate the onset of collagen type II-induced arthritis in mice

**DOI:** 10.1186/ar3814

**Published:** 2012-04-26

**Authors:** Rob J Vandebriel, Hilda JI De Jong, Eric R Gremmer, Olaf H Klungel, Jan-Willem Cohen Tervaert, Wout Slob, Jan Willem Van Der Laan, Henk Van Loveren

**Affiliations:** 1 Laboratory for Health Protection Research, National Institute for Public Health and the Environment, Antonie van Leeuwenhoeklaan 9, PO Box 1, 3720 BA Bilthoven, the Netherlands; 2 Department of Toxicogenomics, Maastricht University, Universiteitssingel 50, PO Box 616, 6200 MD Maastricht, the Netherlands; 3 Division of Pharmacoepidemiology and Clinical Pharmacology, Utrecht Institute for Pharmaceutical Sciences, Utrecht University, Universiteitsweg 99, PO Box 80 082, 3508 TB Utrecht, the Netherlands; 4 Department of Internal Medicine, Division of Clinical and Experimental Immunology, University Hospital Maastricht, Universiteitssingel 50, PO Box 616, 6200 MD Maastricht, the Netherlands; 5 Centre for Substances and Integrated Risk Assessment, National Institute for Public Health and the Environment, Antonie van Leeuwenhoeklaan 9, PO Box 1, 3720 BA Bilthoven, the Netherlands; 6 Section on Pharmacology, Toxicology and Biotechnology (FTBB), Medicines Evaluation Board, Graadt van Roggenweg 500, PO Box 8275, 3503 RG Utrecht, the Netherlands

## Abstract

**Introduction:**

Statins (hydroxymethylglutaryl coenzyme A reductase inhibitors) are effective in reducing the risk of cardiovascular morbidity and mortality in patients with hyperlipidemia, hypertension, or type II diabetes. Next to their cholesterol-lowering activity, statins have immunomodulatory properties. Based on these properties, we hypothesized that statin use may eventually lead to dysregulation of immune responses, possibly resulting in autoimmunity. We have recently shown in an observational study that statin use was associated with an increased risk of developing rheumatoid arthritis. Our objective was to investigate whether a causal relationship could be established for this finding.

**Methods:**

The mouse collagen type II (CII)-induced arthritis (CIA) model was used, with immunization, challenge, and euthanasia at days 0, 21, and 42, respectively. Statins were given orally before (day -28 until day 21) or after (day 21 until day 42) CIA induction. Atorvastatin (0.2 mg/day) or pravastatin (0.8 mg/day) was administered. Arthritis was recorded three times a week. Serum anti-CII autoantibodies and cytokines in supernatants from Concanavalin-A-stimulated lymph node cells and CII-stimulated spleen cells were measured.

**Results:**

Statin administration accelerated arthritis onset and resulted in 100% arthritic animals, whereas only seven out of 12 nonstatin control animals developed arthritis. Atorvastatin administration after CIA induction resulted in earlier onset than atorvastatin administration before induction, or than pravastatin administration before or after induction. The arthritic score of animals given pravastatin before CIA induction was similar to that of the nonstatin controls, whereas the other groups that received statins showed higher arthritic scores. Atorvastatin administration, especially before CIA induction, increased anti-CII autoantibody production. IL-2 and IL-17 production by lymph node and spleen cells was higher in CIA animals than in PBS controls, but was not affected by statin administration. While IFNγ production was not affected by CIA induction, atorvastatin administration before CIA induction increased the production of this cytokine.

**Conclusion:**

These data support previous results from our observational studies, indicating a role for statins in the induction of autoimmunity.

## Introduction

Statins (hydroxymethylglutaryl coenzyme A reductase inhibitors) have been shown to be effective in reducing the risk of cardiovascular morbidity and mortality in patients with hyperlipidemia, hypertension, or type II diabetes [1-4]. In addition to their cholesterol-lowering activity, several studies have shown that statins have anti-inflammatory and immunomodulatory properties [5-8]. We hypothesized that because of these immunomodulatory properties statin use may eventually lead to dysregulation of immune responses, possibly resulting in autoimmunity. In line with this hypothesis, we have recently shown in an observational study that statin use was associated with an increased risk of developing rheumatoid arthritis (RA) [9]. Moreover, based on individual case reports we found a positive association between statin use and the occurrence of lupus-like syndrome [10]. This latter association was recently confirmed by another research group [11].

To investigate whether a causal relationship can be established for these observations, we evaluated the effects of statin administration on arthritis in the collagen type II (CII)-induced arthritis (CIA) mouse model. In this model for RA, mice are immunized with CII mixed with Freund's complete adjuvant, and are challenged 3 weeks later with CII alone. Arthritis is scored from the time of challenge onwards [12].

Several studies have shown beneficial effects of statin administration on joint inflammation in the mouse CIA model [13-16]. However, these studies did not specifically address the effects of statin administration before arthritis induction, an issue that follows from our observational study [9]. Nonetheless, to relate our results to the animal studies indicated above [13-16], we also evaluated effects of statin administration after arthritis induction.

Here we show that statin administration accelerated arthritis onset and resulted in 100% arthritic animals, whereas only seven out of 12 nonstatin control animals developed arthritis. Atorvastatin administration after CIA induction resulted in earlier onset than atorvastatin administration before induction, or than pravastatin administration before or after induction. The arthritic score of animals given pravastatin before CIA induction was similar to that of the nonstatin controls, whereas the other groups that received statins showed higher arthritic scores. Atorvastatin administration, especially before CIA induction, increased anti-CII autoantibody production. IL-2 and IL-17 production by lymph node (LN) and spleen cells was not affected by statin administration. Atorvastatin administration before CIA induction increased IFNγ production.

## Materials and methods

### Induction and assessment of collagen-induced arthritis

Male DBA/1OlaHsd mice were obtained from Harlan (Horst, the Netherlands). At the age of 10 to 12 weeks the animals were injected intradermally at the base of the tail with 100 μl of an emulsion of bovine CII (Chondrex, Redmond, WA, USA) and Freund's complete adjuvant (Chondrex) on day 0 (final concentrations of CII and Freund's complete adjuvant, 1 mg/ml). The mice were challenged by intraperitoneal injection of 100 μl CII (concentration 1 mg/ml) on day 21. Body weight and arthritis severity were assessed three times per week in a blinded manner, using a semiquantitative scoring system (with scores ranging from 0 to 4 for each paw) until the mice were euthanized on day 42 [12]. Arthritis scoring was performed by two individuals, alternating between days; there were no visible systematic differences in the way they scored. Mice were anesthetized with ketamine, rompun, and atropine, and blood was collected from the orbital plexus. Inguinal LNs of the hind paws and the spleens were excised.

The animal experiments were approved prior to their commencement by an independent ethical committee, in accordance with national legislation.

### Statin administration

**Figure 1:**
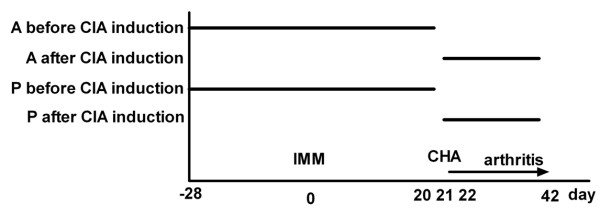
**Scheme of collagen-induced arthritis induction and statin administration**. Mice were immunized at day 0 (IMM) and challenged at day 21 (CHA), after which arthritis was scored three times a week until day 42. Atorvastatin (A) or pravastatin (P) were given daily, either before (day -28 until day 20) or after (day 22 until day 42) collagen-induced arthritis (CIA) induction. Mice were euthanized at day 42.

Atorvastatin (calcium salt) was a kind gift from Pfizer (Groton, CT, USA). Pravastatin (sodium salt) was obtained from Teva (Debrecen, Hungary). Atorvastatin (1 mg/ml) and pravastatin (4 mg/ml) were administered daily by oral gavage (0.2 ml). The atorvastatin dose approximates 10 mg/kg body weight [17]. The lipid-lowering and lipoprotein-lowering efficacy of pravastatin is four times lower than that of atorvastatin [18], and pravastatin was thus given at a four times higher concentration than atorvastatin. Atorvastatin and pravastatin were given either before challenge (day -28 until day 20) or after challenge (day 22 until day 42; see Figure 1). PBS (0.2 ml) was given as a control on days when statins were not administered. One control group received only PBS, and a second control group (negative control) received only PBS and arthritis was not induced. These groups received 0.2 ml PBS by oral gavage daily throughout the experiment. There were 12 mice per group.

### Measurement of autoantibodies

Anti-bovine CII IgG antibodies were measured using an ELISA (mouse anti-bovine CII IgG assay kit; Chondrex) according to the manufacturer's instructions. Briefly, after incubation with blocking buffer, a serial dilution of the standard as well as a 20,000-fold dilution of serum samples was incubated. After incubation with peroxidase-conjugated goat anti-mouse IgG, *O*-phenylenediamine dihydrochloride was added, the reaction was stopped, and the optical density was measured at 490 nm. The plates were washed before and between all incubation steps.

### Cell culture

The culture medium used was RPMI-1640 (Gibco, Grand Island, NY, USA) supplemented with 10% FCS, 100 μg/ml streptomycin, and 100 IU/ml penicillin. Cell suspensions were made by pressing the LNs and spleens through a cell strainer (Falcon, Franklin Lakes, NJ, USA). Cells were counted using a Coulter Counter (Coulter Electronics, Luton, UK). LN cell suspensions were cultured at 10^6 ^cells/ml culture medium with 5 μg/ml Concanavalin A (MP Biomedicals, Irvine, CA, USA) in 96-well tissue culture plates (Nunc, Roskilde, Denmark) for 24 hours. Spleen cell suspensions were cultured at 10^6 ^cells/ml culture medium with 50 μg/ml CII or 5 μg/ml Concanavalin A in 96-well tissue culture plates (Nunc) for 72 hours. Culture conditions were 37°C in a humidified atmosphere containing 5% carbon dioxide.

### Cytokine measurements

A 10-plex panel containing beads for mouse IL-1*, IL-2, IL-4, IL-6, IL-10, IL-12p70, IL-17, granulocyte-macrophage colony-stimulating factor, IFNγ, and TNF* (Bio-Rad, Hercules, CA, USA) was used. After incubation and washing steps [19], the beads were measured on a Bio-Plex (Bio-Rad).

### Statistical analysis of arthritis induction

We analyzed the fraction of animals with arthritis as a function of time, by fitting time-response models to our data. We used PROAST, a general program for dose-response modeling [20,21]. These models apply directly by regarding time rather than dose as the independent variable - that is, the time of onset of arthritis defined as the median time when 50% of the animals develop arthritis (ET_50_)). The fitted model was the log-logistic model:

$$y=a+\left(1-a\right)/\left\{1+\text{exp}\left[c\;\text{ln}\left(b/x\right)\right]\right\}$$

where *y *is the fraction of animals showing arthritis and *x *is the time (*a, b *and *c *are constants). First, the model was fitted to the data from all (five) experimental groups combined - that is, the same ET_50 _value was estimated for all treatment groups. The same model was then fitted but now by estimating a treatment-specific ET_50 _value. When the associated fit of the model (as reflected by the value of the log-likelihood) is significantly improved (likelihood ratio test), this indicates a significant treatment effect on the ET_50 _value.

The analysis of the fraction of animals with arthritis over time as described above is statistically invalid because the observations over time relate to the same animals (that is, the assumption of independent observations does not hold). As a result, *P *values and confidence intervals of the means will be too optimistic. We therefore performed an additional analysis as follows. The arthritis severity scores were analyzed as a function of time using a so-called latent-variable model. In this model the latent variable reflects the arthritis severity as a continuously increasing value with time, while the observed scores are imagined to arise from consecutive cutoff values on that underlying continuous severity scale. These cutoff values are estimated while fitting the time-response model to the scores [22]. The latent-variable model:

$$y=a\;\text{exp}\left(b\times d\right)$$

where *y *is the latent variable and *x *is time (*a, b *and *d *are constants), was fitted to the observed scores for all individuals combined, by including the factor individual animal as a covariate. Individuals were found to significantly differ in the time-response curve regarding parameter *b*, but parameters *a *and *d *were not found to differ significantly. Hence, we fitted the model with only parameter *b *dependent on the individual animal. The resulting estimates for *b *were then analyzed in a one-way analysis of variance to compare the treatments. In this analysis the estimates for *b *are statistically independent. Because the results of statin treatments on arthritis induction using the latent variable model were similar to the results using the log-likelihood model, we have chosen to show the results of the latter model.

### General statistical analysis

The effect of arthritis induction on body weight, autoantibody production, and cytokine production was assessed using an independent-samples *t *test (SPSS Inc., Chicago, IL, USA). The effect of statin treatments on body weight, autoantibody production and cytokine production was assessed using one-way analysis of variance, followed by the Bonferroni *post hoc *test (SPSS Inc.).

## Results

### Body weight

**Figure 2:**
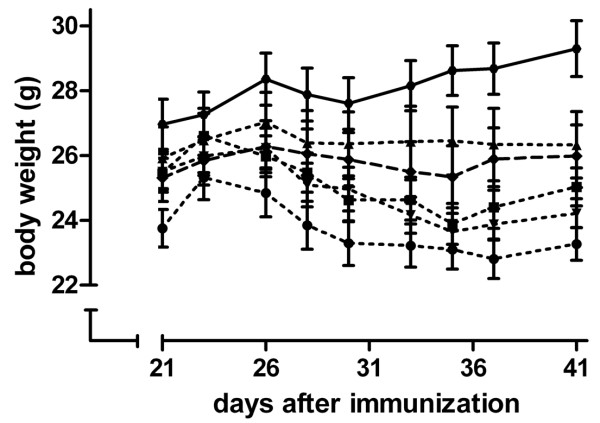
**Effect of collagen-induced arthritis induction and statin treatment on body weight**. Mice were immunized at day 0, challenged at day 21, and weighed three times a week until euthanasia at day 42. Statins were given daily. Black circles connected by dotted line, atorvastatin before collagen-induced arthritis (CIA) induction; black squares connected by dotted line, atorvastatin after CIA induction; black triangles connected by dotted line, pravastatin before CIA induction; black inverted triangles connected by dotted line, pravastatin after CIA induction; black diamonds connected by dotted line, CIA control; black circles connected by continuous line, negative control. *n *= 12 per group. The effect of arthritis induction was assessed using an independent-samples *t *test; the effect of statin treatments was assessed using one-way analysis of variance, followed by the Bonferroni *post hoc *test.

Induction of CIA resulted in a lower body weight compared with negative control animals (Figure 2). This difference was statistically significant (*P *< 0.05) from day 33 after immunization onwards. Atorvastatin administration before CIA induction resulted in a lower body weight compared with CIA control animals (Figure 2). This difference was statistically significant (*P *< 0.05) from day 37 after immunization onwards. Atorvastatin administration after CIA induction, or pravastatin administration before or after CIA induction, did not significantly alter body weight compared with CIA control animals.

### Arthritis

**Figure 3:**
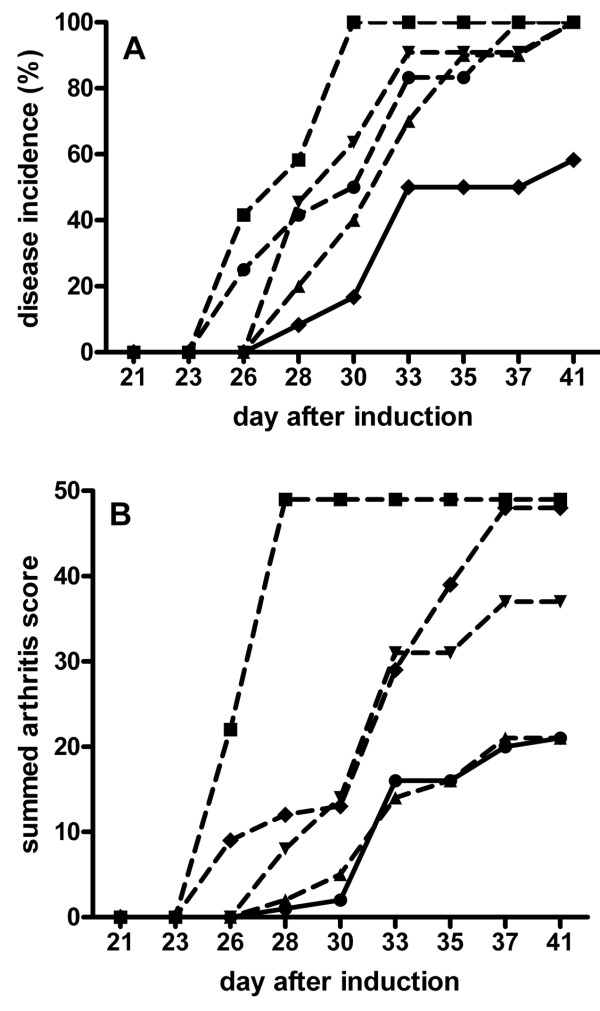
**Effect of statin treatment on arthritis incidence and score**. **(A) **Number of arthritic animals per group versus time after immunization. **(B) **Summed arthritis score per group of animals versus time after immunization. Mice were immunized at day 0 and challenged at day 21, after which arthritis was scored three times a week until day 42. Statins were given daily. Black circles connected by dotted line, atorvastatin before collagen-induced arthritis (CIA) induction; black squares connected by dotted line, atorvastatin after CIA induction; black triangles connected by dotted line, pravastatin before CIA induction; black inverted triangles connected by dotted line, pravastatin after CIA induction; black diamonds connected by continuous line, CIA control. *n *= 12 per group.

**Figure 4:**
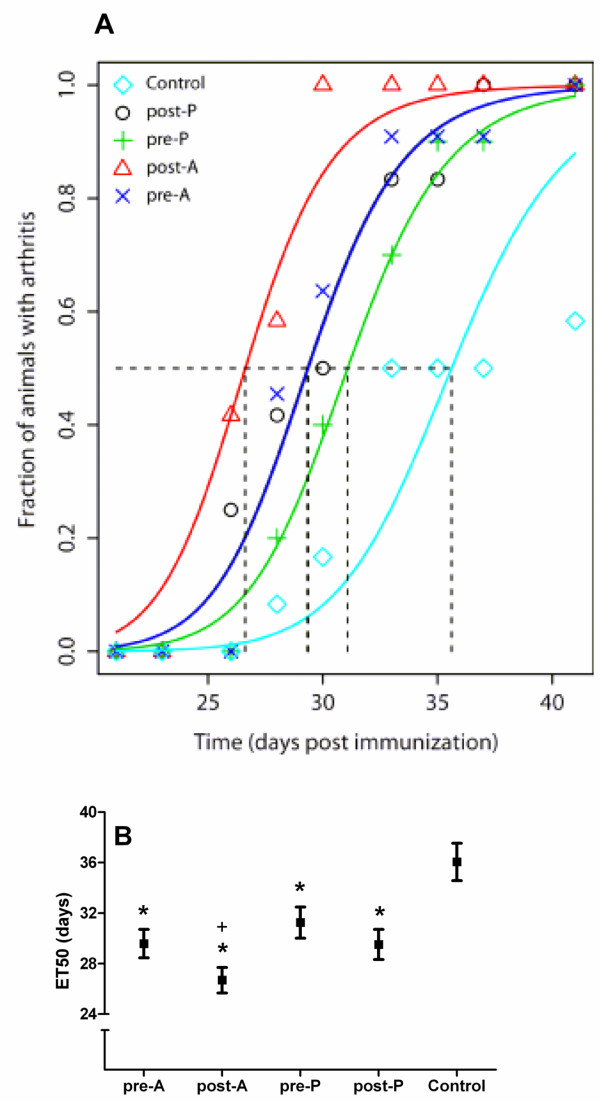
**Statin administration accelerates arthritis onset (fraction of arthritic animals versus time)**. Mice were immunized at day 0 and challenged at day 21, after which arthritis was scored three times a week until day 42. **(A) **Mice were given atorvastatin before challenge (day -28 until day 20, pre-A; cross sign, dark blue line) or after challenge (day 22 until day 42, post-A; open triangle, red line), or they were given pravastatin before challenge (pre-P; plus sign, green line) or after challenge (post-P; open circle, black line). Control mice (open diamond, light blue line) did not receive statins. The dark blue and black lines are overlapping. **(B) **Median time when 50% of the animals develop arthritis (ET_50 _values; mean and 90% confidence intervals), calculated from (A). *Significantly different (*P *< 0.05) from control mice; ^+^significantly different (*P *< 0.05) from pre-A, pre-P, and post-P treated mice. *n *= 12 per group. ET_50 _values (mean and 90% confidence intervals) were calculated using the PROAST program [20,21]. One-way analysis of variance, followed by the Bonferroni *post hoc *test.

Statin administration (both atorvastatin and pravastatin, both before and after CIA induction) resulted in 100% arthritic animals at the time of euthanasia, whereas only seven out of 12 nonstatin control animals had developed arthritis at that time point (Figure 3A). Statin administration resulted in earlier arthritis onset, expressed as the day after immunization in which 50% of the animals show arthritis (Figure 4). Atorvastatin administration after CIA induction resulted in earlier onset than atorvastatin administration before CIA induction, or than pravastatin administration before or after CIA induction (Figure 4).

The arthritic score summed per group of animals showed a similar score for the animals that received pravastatin before CIA induction and nonstatin controls, whereas the other groups of statin-administered animals showed higher scores (Figure 3B). Only seven out of 12 nonstatin controls became arthritic, so the mean arthritic score for animals that received pravastatin before CIA induction was lower than for the nonstatin controls (data not shown).

### Autoantibody production

**Figure 5:**
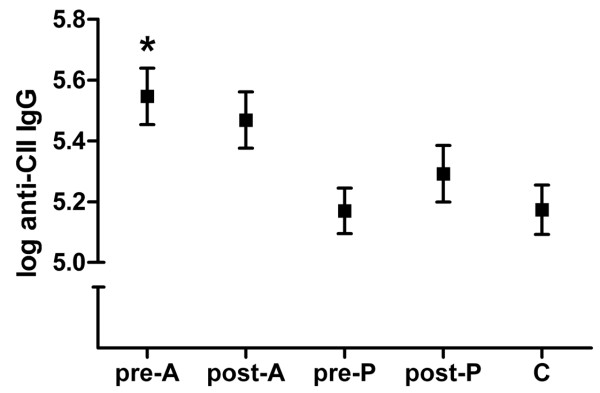
**Anti-collagen type II IgG levels**. Mice were immunized at day 0 and challenged at day 21. Atorvastatin (A) or pravastatin (P) were given daily, either before (day -28 until day 20) or after (day 22 until day 42) collagen-induced arthritis induction. Mice were euthanized at day 42. Control mice (C) did not receive statins. Anti-collagen type II (anti-CII) IgG was measured in serum by ELISA. *Significantly different (*P *< 0.05) from C. *n *= 12 per group. One-way analysis of variance, followed by the Bonferroni post hoc test.

Induction of CIA resulted in the presence of anti-CII antibodies (log IgG level = 5.17; *P *< 0.001 compared with the negative controls). Atorvastatin administration resulted in higher anti-CII antibody production compared with pravastatin administration and the CIA control (*P *= 0.005 and *P *= 0.013, respectively). In addition, atorvastatin administration before CIA induction increased the anti-CII antibody production compared with the CIA control (*P *< 0.05; Figure 5).

### Cytokine production

**Figure 6:**
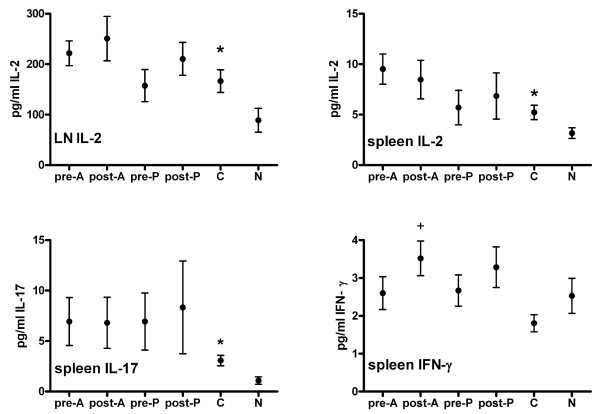
**Cytokine production by lymph node and spleen cells**. **(A) **IL-2 production by Concanavalin-A (Con A)-stimulated inguinal lymph node (LN) cells. **(B) **IL-2 production by collagen type II (CII)-stimulated spleen cells. **(C) **IL-17 production by CII-stimulated spleen cells. **(D) **IFNγ production by CII-stimulated spleen cells. Mice were immunized at day 0 and challenged at day 21. Atorvastatin (A) or pravastatin (P) were given daily, either before (day -28 until day 20) or after (day 22 until day 42) collagen-induced arthritis induction. Mice were euthanized at day 42. Control mice (C) did not receive statins. In negative mice (N), arthritis was not induced and these mice did not receive statins. Inguinal LNs were excised and LN cells were cultured in the presence of Con A for 24 hours. Spleens were excised and spleen cells were cultured in the presence of CII for 72 hours. Cytokine content was measured in the supernatants. *n *= 12. *Significantly different (*P *< 0.05) from N; ^+^significantly different (*P *< 0.05) from C. *n *= 12 per group. The effect of arthritis induction was assessed using an independent-samples *t *test; the effect of statin treatments was assessed using one-way analysis of variance, followed by the Bonferroni *post hoc *test.

Induction of CIA resulted in increased IL-2 production by Concanavalin-A-stimulated LN cells and increased IL-2 and IL-17 production by CII-stimulated spleen cells (*P *< 0.05; Figure 6). For none of the other cytokines tested was a significant effect due to CIA induction found.

Atorvastatin administration after CIA induction increased IFNγ production by CII-stimulated spleen cells (*P *< 0.05; Figure 6). Combining the results of both statins, administration after but not before CIA induction resulted in increased production (*P *< 0.01). Combining the results of both time points, both atorvastatin and pravastatin increased IFNγ production (*P *< 0.05). For none of the other cytokines tested was a significant effect due to statin administration found.

## Discussion

Here we have shown that statin administration results in 100% arthritic animals, whereas only seven out of 12 nonstatin control animals developed arthritis. Moreover, statin administration accelerated arthritis onset. This accelerated onset was seen when statin administration was started either before or after CIA induction. Accelerated onset resulting from statin administration before CIA induction is in line with our hypothesis of statin-induced immune deviation resulting in autoimmunity [9,10]. In addition, we observed that atorvastatin administration after CIA induction resulted in earlier arthritis induction than administration with the same statin before CIA induction. Overall, atorvastatin had a somewhat stronger effect on accelerating onset than pravastatin. Atorvastatin administration before or after CIA induction, or pravastatin administration after CIA induction, resulted in a similar mean arthritic score compared with the nonstatin control, while pravastatin administration before CIA induction resulted in a lower mean arthritic score. In this latter pravastatin group the acceleration of arthritis onset was 5 days, while that in the other cases was 7 or 9 days. This lower mean arthritic score may suggest a beneficial effect of pravastatin. This dosing regime, however, also results in accelerated arthritis onset and an increased number of arthritic animals.

Other studies using the mouse CIA model have shown beneficial effects of statin administration on joint inflammation [13-16]. To establish whether statin type, dose, route of administration, or timing of administration relative to CIA induction influenced the clinical outcome, the results of the above-mentioned studies as well as our data are compared in Table 1. Suppression of arthritis development was seen after oral administration of 10 mg/kg atorvastatin (the same exposure route and dose as we used in our study) during (almost) the whole period from immunization to euthanasia [13]. This finding contrasts our results that show accelerated onset when 10 mg/kg atorvastatin was given orally either before or after CII challenge. This issue needs to be resolved in future studies. Suppression of arthritis development was also found after intraperitoneal administration of 100 mg/kg pravastatin [14] or 40 mg/kg simvastatin [15,16]. Using other statins, lower doses, or other exposure routes failed to show an effect on arthritis. An accelerated onset of arthritis was only found in our study.

**Table 1:** Comparison of statin effects on murine collagen-induced arthritis

Study	Statin	Dose (mg/kg)	Exposure route	Timing (days)	Effect on arthritis
Present study	A	10	Oral	-28 to 20	ΔET_50 _-7 days
Present study	A	10	Oral	22 to 42	ΔET_50 _-9 days
Present study	P	40	Oral	-28 to 20	ΔET_50 _-5 days
Present study	P	40	Oral	22 to 42	ΔET_50 _-7 days
Ho and colleagues [13]	A	10	Oral	2 to 49	Suppression
Yamagata and colleagues [14]	P	100	Intraperitoneal	0 to 35	Suppression
Leung and colleagues [15]	S	40	Intraperitoneal	21 to 40	Suppression
Leung and colleagues [15]	S	20	Intraperitoneal	21 to 40	No effect
Leung and colleagues [15]	S	10	Intraperitoneal	21 to 40	No effect
Palmer and colleagues [16]	A	100	Oral	0 to 48	No effect
Palmer and colleagues [16]	A	1	Oral	0 to 48	No effect
Palmer and colleagues [16]	R	2	Subcutaneous	0 to 48	No effect
Palmer and colleagues [16]	R	0.2	Subcutaneous	0 to 48	No effect
Palmer and colleagues [16]	R	40	Intraperitoneal	21 to 35	No effect
Palmer and colleagues [16]	S	40	Intraperitoneal	21 to 35	Suppression
Palmer and colleagues [16]	S	40	Oral	21 to 35	No effect

A, atorvastatin; P, pravastatin; S, simvastatin; R, rosuvastatin; ΔET_50_, number of days between the time when 50% of the animals developed arthritis (ET_50 _value) for the statin-treated animals and the ET_50 _value for the control animals.

Atorvastatin administration before CIA induction resulted in increased anti-CII antibody production, whereas atorvastatin administration after CIA induction resulted in a nonsignificant increase in production of this antibody. Pravastatin administration before and after CIA induction, however, did not affect anti-CII IgG antibody production.

Atorvastatin administration before CIA induction resulted in accelerated arthritis onset and increased autoantibody production. This observation may suggest that increased autoantibody production plays a role in accelerated arthritis onset. Our observational study showed that statin use is associated with an increased risk of developing RA [9]. In humans, IgM rheumatoid factor and anti-cyclic citrullinated peptide antibodies have been found years before RA becomes clinically apparent [23]. For RA patients that used statins before developing clinically manifest disease, therefore, statin use may have coincided with autoantibody development. Statins may be speculated to increase autoantibody production, thereby facilitating RA development.

CIA induction resulted in increased IL-2 production by Concanavalin-A-stimulated LN cells, probably reflecting the stronger activation or higher frequency of T cells in the LN draining the inflamed joints. Production of this cytokine was not significantly affected by statin administration. Similarly, CIA induction resulted in increased IL-2 production by CII-stimulated spleen cells, and production of IL-2 was not significantly affected by statin administration.

Furthermore, CIA induction resulted in increased IL-17 production by CII-stimulated spleen cells. Since IL-23-deficient mice that lack IL-17-producing CD4^+ ^cells (Th17 cells) are resistant to CIA induction [24], Th17 cells are assumed to play an essential role in CIA. The activation or frequency of Th17 cells in the spleen is probably increased in arthritic mice, in line with previous studies [24-27]. Importantly, IL-17 production was not significantly affected by statin administration.

In addition, CIA induction resulted in decreased IFNγ production by LN cells (0.75-fold) and CII-stimulated spleen cells (0.71-fold), albeit not statistically significantly (data not shown). Since IFNγ acts as a disease-limiting factor in CIA [28], reduced levels of this cytokine may possibly be characteristic for CIA. Since atorvastatin was shown to inhibit IFNγ production [29], and absence of IFNγ signaling resulted in accelerated arthritis onset [30,31], reduced production of this cytokine may be a possible mechanism underlying accelerated arthritis onset due to atorvastatin treatment. We did not, however, observe altered IFNγ production due to statin administration. Taking together the IL-17 and IFNγ data, the increased IL-17 and decreased IFNγ production upon CIA induction fits the selective induction of Th17 and not T-helper type 1 cells in CIA [24].

Another possible mechanism by which statins may affect arthritis is through their induction of caspase-1, IL-1β, and IL-18 in monocytes, resulting in increased IFNγ production by T cells [32]. Interestingly, this increased IFNγ production occurred only at low statin concentrations. Caspase-1 activation results in cleavage of pro-IL-1β and pro-IL-18 to their mature (active) forms. Caspase-1, IL-1β, and IL-18 are all implicated in CIA. The pharmacologic caspase-1 inhibitor VX-765 reduced paw inflammation when given as a prophylactic and a therapeutic [33]. IL-1β administration accelerated arthritis [34], while anti-IL-1β treatment markedly suppressed established arthritis [35]. IL-18 administration resulted in higher arthritis incidence and severity [36,37], while IL-18-deficient mice showed reduced arthritis incidence and severity [38]. This observation may suggest that statins aggravate arthritis by activating caspase-1, thereby inducing IL-1β and IL-18. Increased IFNγ production may, on the contrary, reduce arthritis. Taking the two possible mechanisms together, caspase-1 activation by statins may aggravate arthritis by inducing IL-1β and IL-18. The resulting IFNγ induction that in itself reduces arthritis may be counteracted by the T-helper type 2 skewing effect of statins. Clearly, disease outcome results from a complicated interplay between various effects of statins. In our hands, statin exposure resulted in accelerated arthritis onset.

In summary, the anti-CII antibody, IL-2, IL-17, and IFNγ data do not provide a possible mechanism for the accelerated arthritis observed in this study. It should be noted, however, that establishing such a mechanism was not the aim of our study. We aimed to investigate whether a causal relationship could be established for the association between statin use and an increased risk of developing RA.

The CIA model was chosen because our observational study has shown that statin use was associated with an increased risk of developing RA [9]; because it is a model for RA, recapitulating many aspects of the disease [39-42]; because an induced model is preferred to a spontaneous model, since only in an induced model can the effects of statin use prior to expression of arthritis be tested; and because the mouse CIA model is both B-cell and T-cell mediated and is therefore more similar to human RA than models such as the rat adjuvant arthritis model, which is (only) T-cell mediated.

## Conclusions

Here we have shown that administration of atorvastatin and pravastatin resulted in accelerated arthritis onset in a mouse CIA model. Atorvastatin administration after CIA induction resulted in earlier onset than atorvastatin administration before CIA induction, or than pravastatin administration before or after CIA induction. Atorvastatin administration, especially before CIA induction, resulted in increased autoantibody production. Importantly, our data provide a causal relationship for previous results obtained from observational studies [9,10].

## Abbreviations

CIA: collagen-induced arthritis; CII: collagen type II; ELISA: enzyme-linked immunosorbent assay; ET_50_: median time when 50% of the animals develop arthritis; FCS: fetal calf serum; IFN: interferon; IL: interleukin; LN: lymph node; PBS: phosphate-buffered saline; RA: rheumatoid arthritis; Th17: T-helper type 17 cells; TNF: tumor necrosis factor.

## Competing interests

The authors declare that they have no competing interests.

## Authors' contributions

RJV, J-WCT, WS, JWVDL, and HVL designed the study. RJV and ERG performed the experiments. RJV and WS performed the statistical analysis. RJV, WS, and HVL analyzed and interpreted the data. RJV, HJIDJ, OHK, J-WCT, WS, JWVDL, and HVL prepared the manuscript. All authors read and approved the final manuscript.
